# Combinatorial *In Silico* Strategy towards Identifying Potential Hotspots during Inhibition of Structurally Identical HDAC1 and HDAC2 Enzymes for Effective Chemotherapy against Neurological Disorders

**DOI:** 10.3389/fnmol.2017.00357

**Published:** 2017-11-09

**Authors:** Shabir Ahmad Ganai, Ehsaan Abdullah, Romana Rashid, Mohammad Altaf

**Affiliations:** Chromatin and Epigenetics Lab, Department of Biotechnology, University of Kashmir, Srinagar, India

**Keywords:** HDAC2, HDACi, molecular docking, MM-GBSA, MDS, e-Pharmacophores, neurodegenerative disorders, e-Pharmacophores based virtual screening

## Abstract

Histone deacetylases (HDACs) regulate epigenetic gene expression programs by modulating chromatin architecture and are required for neuronal development. Dysregulation of HDACs and aberrant chromatin acetylation homeostasis have been implicated in various diseases ranging from cancer to neurodegenerative disorders. Histone deacetylase inhibitors (HDACi), the small molecules interfering HDACs have shown enhanced acetylation of the genome and are gaining great attention as potent drugs for treating cancer and neurodegeneration. HDAC2 overexpression has implications in decreasing dendrite spine density, synaptic plasticity and in triggering neurodegenerative signaling. Pharmacological intervention against HDAC2 though promising also targets neuroprotective HDAC1 due to high sequence identity (94%) with former in catalytic domain, culminating in debilitating off-target effects and creating hindrance in the defined intervention. This emphasizes the need of designing HDAC2-selective inhibitors to overcome these vicious effects and for escalating the therapeutic efficacy. Here we report a top-down combinatorial *in silico* approach for identifying the structural variants that are substantial for interactions against HDAC1 and HDAC2 enzymes. We used extra-precision (XP)-molecular docking, Molecular Mechanics Generalized Born Surface Area (MMGBSA) for predicting affinity of inhibitors against the HDAC1 and HDAC2 enzymes. Importantly, we employed a novel *in silico* strategy of coupling the state-of-the-art molecular dynamics simulation (MDS) to energetically-optimized structure based pharmacophores (e-Pharmacophores) method via MDS trajectory clustering for hypothesizing the e-Pharmacophore models. Further, we performed e-Pharmacophores based virtual screening against phase database containing millions of compounds. We validated the data by performing the molecular docking and MM-GBSA studies for the selected hits among the retrieved ones. Our studies attributed inhibitor potency to the ability of forming multiple interactions and infirm potency to least interactions. Moreover, our studies delineated that a single HDAC inhibitor portrays differential features against HDAC1 and HDAC2 enzymes. The high affinity and selective HDAC2 inhibitors retrieved through e-Pharmacophores based virtual screening will play a critical role in ameliorating neurodegenerative signaling without hampering the neuroprotective isoform (HDAC1).

## Introduction

Chromatin architecture plays a decisive role in transcriptional regulation which in turn is potentially modulated by the antagonistic activity of HATs and Histone deacetylases (HDACs) (Eberharter and Becker, 2002; Qi et al., 2011). While HATs favor the transcriptional activation via chromatin decondensation, HDACs promote chromatin condensation and subsequent gene silencing (Yang and Seto, 2007). The opposing activities of HATs and HDACs regulate acetylation homeostasis that plays a crucial role in governing various gene expression programs (Ropero and Esteller, 2007; Ganai, 2016a). HDACs are conjugated enzymes modulating both histone and non-histone substrates and act as corepressors in transcriptional events (Yang and Seto, 2008; Mottamal et al., 2015; Ganai, 2016b). The 18 HDACs identified in human beings till date, have been divided into four classes based on structural resemblance to yeast HDACs (Mottamal et al., 2015). Class I HDACs mainly lack shuttling ability, are ubiquitous in distribution and includes HDAC1, HDAC2, HDAC3 and HDAC8. Unlike Class I HDACs, Class II HDACs are tissue specific in distribution and possess shuttling ability (Fischle et al., 2001). This Class is further subdivided into Class IIa and Class IIb HDACs. While Class IIa covers HDAC4, 5, 7, and 9, Class IIb encompasses HDAC6 and HDAC10 (Ganai, 2017a). Class III includes mechanistically distinct HDACs termed as Sirtuins (SIRT1-SIRT7) (Albani et al., 2010; Morris et al., 2010). Class IV includes HDAC11 as the only member and in comparison to other HDACs is least studied (Seto and Yoshida, 2014). Class I, II and Class IV HDACs are Zinc (Zn^2+^) dependent and are also known as classical HDACs. Sirtuins are NAD^+^ dependent and are associated with cellular senescence and aging (Ganai, 2016c; Watroba and Szukiewicz, 2016). Among Class I HDACs, HDAC1 and HDAC2 show the highest structural identity with each other.

Histone deacetylase inhibitors (HDACi), the small molecules interfering HDACs are emerging as potent chemotherapeutic agents. Based on structural distinction these inhibitors may be hydroxamates like suberoylanilide hydroxamic acid (SAHA), Trichostatin A (TSA); benzamide derivatives like pyridin-3-ylmethyl N-[[4-[(2-aminophenyl) carbamoyl] phenyl]methyl]carbamate (MS-275 or entinostat), 4-acetamido-N-(2-aminophenyl)benzamide (CI-994); cyclic peptides like romidepsin and (3S,6R,9S,12R)-6,9-dimethyl-3-[6-(oxiran-2-yl)-6-oxohexyl]-1,4,7,10-tetrazabicyclo[10.3.0]pentadecane-2,5,8,11-tetrone (HC-toxin); short chain fatty acids encompassing sodium butyrate, phenylbutyrate and valproic acid. These inhibitors have shown promising activity against cancer and neurodegeneration. Four HDACi are approved by US-FDA for treating distinct malignancies. SAHA has been approved for treating cutaneous T-cell lymphoma (CTCL). Romidepsin followed SAHA in gaining approval and is currently used against CTCL and peripheral T-cell lymphoma (PTCL) (Ververis et al., 2013). Belinostat, the third HDAC inhibitor has been approved for relapsed/refractory PTCL (Ververis et al., 2013; Ganai, 2016a). The fourth approved inhibitor panobinostat is currently active against multiple myeloma (Ganai, 2016c). Most of the HDACi including vorinostat are pan-inhibitors targetting HDACs of different classes (Dasmahapatra et al., 2010), few like entinostat are class selective targetting isoforms of a given class (Duque-Afonso et al., 2011) and very few like tubacin are isoform selective (Lee et al., 2015; Ganai, 2016d) targetting a single HDAC.

Histone deacetylase inhibitors (HDACi) including LAQ824 (dacinostat), pyroxamide, HC-toxin are composed of three distinct components; Zinc binding group (ZBG) which chelates Zinc (Zn) ion situated deep in the active site; Linker region connecting ZBG with cap region and interacting with active site tunnel residues; Cap region which closes the active site gate and interacts with active site rim residues. This three component concept has proved spectacular in developing potent inhibitors against HDACs. For designing isoform-selective inhibitors specific modifications in these components have been exploited (Witt et al., 2009; Ganai, 2016d, 2017a).

### Implications of HDAC2 in neurodegenerative events

The cellular imbalance between HAT and HDAC activity alters acetylation homeostasis causing transcriptional dysregulation which in turn provides impetus to neurodegenerative signaling (Saha and Pahan, 2006). Aberrant expression of HDACs has been implicated in neurologic pathologies (Ganai et al., 2016). For instance, HDAC6 overexpression has been reported to inhibit nerve growth by deacetylating tubulin protein (Rivieccio et al., 2009). Knockout studies in mouse models have revealed HDAC2 as a key regulator of associative and spatial memory (Guan et al., 2009). While HDAC2 overexpression has been reported to impair memory performance, HDAC2 knockout mice showed improved memory performance (Guan et al., 2009; Volmar and Wahlestedt, 2015). Robust improvement in associative learning has been demonstrated on selective knockout of HDAC2 (Morris et al., 2013). Upregulated HDAC2 levels have been implicated in restraining the expression of neuroplasticity genes during neurodegeneration (Gräff et al., 2012). Mounting evidences suggest that HDAC2 plays a central role in mediating cognitive impairment (Welberg, 2012). Further, the impact of elevated levels of HDAC2 on basic excitatory neurotransmission has been well demonstrated in mature neurons indicating its role in synaptic plasticity (Akhtar et al., 2009). HIV-Tat protein induced upregulation of HDAC2 culminates in down-regulation of genes meant for synaptic plasticity and neuronal function thereby triggering HIV-associated neurocognitive disorders (HAND) (Saiyed et al., 2011). These findings establish the critical role of HDAC2 in modulating synaptic plasticity and enduring changes of neural circuits culminating in negative regulation of learning and memory. Taken together, HDAC2 is a propitious and eye catching epigenetic target for tackling neurodegenerative maladies (d'Ydewalle et al., 2012; Ganai, 2017b).

### Current impediment in targetting HDAC2 for neurological disorders

**Figure 1:**
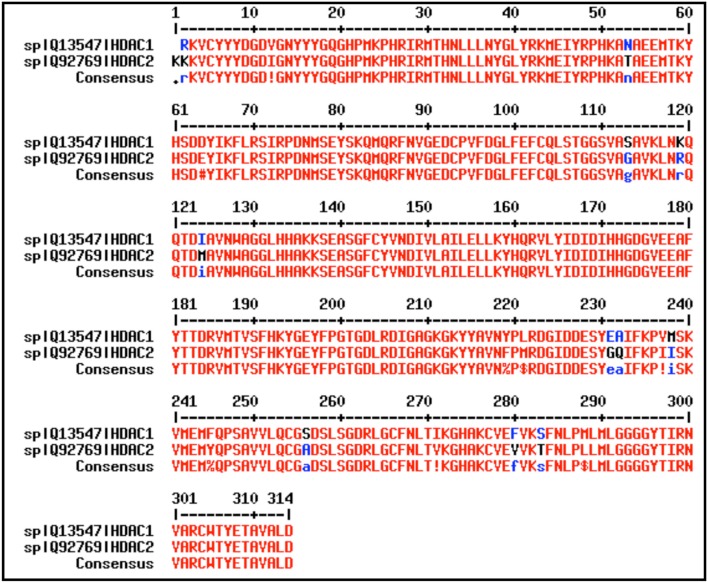
HDAC1 and HDAC2 share high sequence identity (94%) at the active site. The active site residues were taken from UniProt and alignment was performed by using MultAlin and cross checked by using Clustal Omega. Percent identity was calculated by Clustal Omega.

Pharmacological intervention with HDACi reversed the reduced synapse number and learning impairment of HDAC2-overexpressing mice (Guan et al., 2009). Glutamate excitotoxicity has been implicated in many neurodegenerative disorders and studies have shown that inhibitors active against HDAC2 and HDAC3 offer neuroprotection against such toxicity in rat models (Durham, 2012). Unlike HDAC2, HDAC1 interacts with HDRP and facilitates neuronal survival (Bardai et al., 2012). Moreover, the catalytic domains of HDAC1 and HDAC2 share high percentage identity (94%) (Figure 1) and thus therapeutic intervention against the latter also targets HDAC1 (Corpet, 1988; Ganai, 2014). This often causes debilitating off-target effects, emphasizing the desperate need of designing on-target (isoform-selective) inhibitors for HDAC2. Recently two inhibitors BRD4884 and BRD6688 demonstrating kinetic selectivity for HDAC2 vs. HDAC1 have been synthesized (Wagner et al., 2015). Another group has used a novel *de novo* reaction-mechanism- based inhibitor design approach toward the discovery of selective inhibitor β-hydroxymethyl chalcone against HDAC2 (Zhou et al., 2015). Taking these facts into consideration the current study used a combinatorial *in silico* approach including extra-precision molecular docking, molecular mechanics generalized born surface area, molecular dynamics simulation (MDS), trajectory clustering and energetically optimized structure based pharmacophore mapping for highlighting the hotspots of inhibitors in the HDAC1 and HDAC2 binding pocket. Five inhibitors belonging to three different structural groups of HDAC inhibitors were docked against HDAC1 and HDAC2 active site. These docked complexes were subjected to MMGBSA for predicting the binding affinities of docked inhibitors. The docked complexes of top scoring inhibitors LAQ824 and HC-toxin were subject to the cutting edge MDS for 5 ns. The MDS output file of docked complexes was used as input for Desmond trajectory clustering. Seven clusters were generated for each protein-ligand complex and the cluster with maximum number of frames (more stability) was considered for creating hypothesis to highlight the critical features of inhibitor inside the active site of HDAC1 and HDAC2 enzymes.

## Materials and methods

### Protein preparation and grid generation

Accurate starting structures are prerequisite for successful structure based modeling. The crystal structures of HDAC1 and HDAC2 (PDB ID: 4BKX and 4LY1 respectively) retrieved from Protein Data Bank (http://www.rcsb.org) (Lauffer et al., 2013; Millard et al., 2013) were prepared using the Protein Preparation Wizard of Schrödinger package (Maestro v11.0) to ensure structural correctness (Sastry et al., 2013; Ganai et al., 2015a,b). In the first step the missing hydrogen atoms were added to crystal structures and proper bond orders were assigned. Moreover, missing side chains and missing loops were filled using the Prime. All the water molecules beyond 5 Å were deleted. In the next step, the redundant protein chains and heteroatoms were deleted. As HDACs require Zinc for their catalytic function so this heteroatom was kept intact (Ganai et al., 2015b; Sinha et al., 2016; Steinbrecher et al., 2017). Moreover, the native ligand in crystal structure of HDAC2 was kept as such and was used for grid generation in the later stage. The third stage involves the refining of protein structures to make them suitable for subsequent steps. During this process, the structures are optimized and the water molecules with <3 hydrogen bonds to non-waters are deleted. This was followed by minimization in which heavy atoms were converged to Root mean square deviation (RMSD) of 0.30 Å. Grid generation was performed using the cocrystallized ligand as centroid in case of HDAC2 (Glide v7.3). However, in case of HDAC1 lacking the cocrystallized ligand, grid generation was done by specifying the residues interacting with active site Zinc (Sastry et al., 2013; Ganai et al., 2015a).

### Ligand preparation

Computational methods like molecular docking require correct 3D molecular models as initiating point. However, many compounds available in compound databases exist as 2D molecular structures and thus accurate 2D−3D conversion is a critical progenitor to computational analysis. LigPrep incorporated in Schrodinger package generates a single, factual, energy minimized 3D molecular structure with correct chiralities from the given input. Besides, it eradicates mistakes in ligands in order to enhance the accuracy of downstream events including molecular docking (Ganai et al., 2015a; Van Den Driessche and Fourches, 2017). LigPrep can optionally produce many structures from single input structure with various ionization states, stereochemistries, tautomers, and ring conformations. The structure files of 5 ligands used in the study were retrieved from PubChem with PubChem CID's provided in Supplementary Figure 1. These ligands were prepared for molecular docking using the predefined LigPrep (Maestro v11.0) (Kalyaanamoorthy and Chen, 2013; Ganai et al., 2015b). Ligands were desalted; metal binding states were generated as their receptors are Zinc dependent enzymes. Protonation states of ligands and the associated energy penalties were predicted using the Epik (Epik v3.8), a computer program based on Hammett and Taft methodology (Shivakumar et al., 2010). The preparation parameters were kept identical for all ligands to avoid any bias arising due to differential ligand preparation.

### Pose validation by self-docking

Among the various methods used for validating the docking programs, the pose validation method is globally used. Crystal structure of HDAC2 with its cocrystallized ligand was prepared using the protein preparation wizard followed by separation of ligand. The ligand was then redocked against the host target using extra-precision (XP) flexible docking protocol and the RMSD was calculated between crystal and redocked pose (Sandor et al., 2010).

### Molecular docking

The molecular docking was performed using the Glide (Grid-based Ligand Docking with Energetics) of Schrödinger package. The ability of Glide to identify hits for lead optimization and guide understanding of critical interactions apart from desolvation effects influencing receptor-ligand binding, has contributed markedly to its epidemic acceptance. The prepared ligands were docked against grid generated receptors HDAC1 and HDAC2 in an extra precision (XP) flexible mode (Glide v7.3) (Friesner et al., 2006). Identical docking conditions were set for both HDACs to avoid any discrepancy due to differential parameters. The GlideScore (GSore) representing affinity of ligands against receptors was obtained from pose viewer file of docked complexes (Kalyaanamoorthy and Chen, 2013; Ganai et al., 2015b).

### Molecular mechanics generalized born surface area (MMGBSA)

The Prime/MMGBSA tool is widely used for estimating the relative binding affinity of various ligands. The binding energy of ligands calculated by MMGBSA is expected to align well with the experimental binding affinity, especially of a congeneric series. Binding free energy calculations of docked complexes was performed using the Prime module of Schrodinger package. The pose viewer files generated in Glide XP docking were used as input for MMGBSA. The binding free energy was calculated in the frozen state and default dielectric constants, solvation model (VSGB) and OPLS3 force field was used (Lyne et al., 2006; Ganai et al., 2015b). Prime MMGBSA performs five fundamental energy calculations; optimized free receptor (Receptor), optimized free ligand (Ligand), Optimized complex (complex) in addition to receptor from optimized complex and ligand from optimized complex. From these energies, the binding free energy is calculated as:

Prime MMGBSA ΔG (Bind) = Complex − Receptor − Ligand
More negative the value of ΔG (Bind), stronger is the binding.

### Generating ligand-protein interaction profile

Ligand protein interaction profile was generated from the pose of ligand showing the highest GlideScore. All the necessary interactions like hydrogen bonding, pi-pi stacking, pi-cation interactions; salt bridges of the HDAC-HDAC inhibitor docked complexes were generated using the default cutoff (4 Å).

### Molecular dynamics simulation

Molecular dynamics simulation (MDS) is a golden computational technique that helps in monitoring ligand receptor stability and compatibility in an elegant manner. MDS was performed by using Desmond software (Bowers et al., 2006), a relatively novel molecular dynamics (MD) engine by D.E. Shaw Research that can run various molecular simulations including standard MD and simulated annealing (Desmond v4.8) (Shivakumar et al., 2010). The setup files were generated using the system builder option of Desmond. HDAC-HDAC inhibitor complexes were solvated using TIP4P water model (Grover et al., 2012). For specifying shape and size of the repeating unit default orthorhombic boundary conditions were set up. The initial box volume for HDAC2-LAQ824 complex was 463,768 Å^3^ and on minimizing, the volume became 416,755 Å^3^. Same parameters for HDAC2-HC-toxin were 468,092 and 419,994 Å^3^ respectively. While these values for HDAC1-LAQ824 were 463,596 and 450,033 Å^3^, HC- toxin-HDAC1 displayed these volumes as 463,612 and 450,144 Å^3^. Individual systems were neutralized by adding proper number of counter ions. While HDAC2-LAQ824 and HDAC1-LAQ824 systems were neutralized by adding 2 chloride (Cl^−^) ions and no ions respectively, HDAC2-HC-toxin and HDAC1-HC-toxin were neutralized by adding 2 chloride ions (Cl^−^) and 1 sodium ion (Na^+^). Thus, prior to actual MDS a solvated system including enzyme-ligand complexes as solute and water molecules with oppositely charged ions as solvent was generated. Simulation was carried out under NPT (constant number of atoms, constant pressure and constant temperature) ensemble for 100 ns using the molecular dynamics option of Desmond. Constant temperature (300 K) and pressure (1.01325 bar) was maintained throughout the simulation utilizing Nosé-Hoover thermostat (Hoover, 1985) and Martina-Tobias-Klein method (Martyna et al., 1994; Guo et al., 2010). Moreover, all the solvated systems were relaxed prior to simulation. Detailed information like protein and ligand RMSD, root mean square fluctuation (RMSF) and ligand interaction profile was generated from the simulation trajectory of ligand-receptor complexes using simulation interaction diagram option of Desmond (Pravin et al., 2015).

### Creating hypothesis using e-pharmacophores method

Both structure-based protein-ligand docking and ligand-based pharmacophore modeling are essential parts of drug discovery. While ligand-based technologies are time saving, the structure-based approaches are relatively time consuming but can yield more diverse actives and provide essential target insights. The e-Pharmacophores method incorporates the beneficial aspects of both ligand and structure based approaches by generating structure based pharmacophores which are energetically optimized (Kalyaanamoorthy and Chen, 2013). These pharmacophores can serve as representatives in rapid screening of huge databases. The e-pharmacophores were generated using the auto e-Pharmacophores tool of Phase module of Schrödinger package (Phase v4.9). These pharmacophores were generated by coupling MDS to e-Pharmacophores method via Desmond trajectory clustering. The trajectory from MDS for each HDAC-HDAC inhibitor complex was separated into seven different clusters using the predefined approach. E-pharmacophores were generated from cluster with maximum number of frames which is directly related to ligand stability in active site.

### e-Pharmacophores based virtual screening

For validating the e-pharmacophore models, LAQ824 was selected as representative. The e-Pharmacophores of HDAC1-LAQ824 and HDAC2-LAQ824 were used as queries separately and screening was performed against phase database possessing millions of small molecules (Phase v4.9) (Dixon et al., 2006; Natarajan et al., 2016). During virtual screening 50 conformers were generated for each molecule and based on fitness and logic top five hits were selected for each e-Pharmacophores. The hits were docked in extra precision flexible mode (Zhou et al., 2007) against the respective receptors and docking scores were calculated. The docked complexes were subjected to Prime MMGBSA for calculating binding free energy values (Singh and Muthusamy, 2013).

## Results and discussion

### RMSD calculation for pose validation

**Figure 2:**
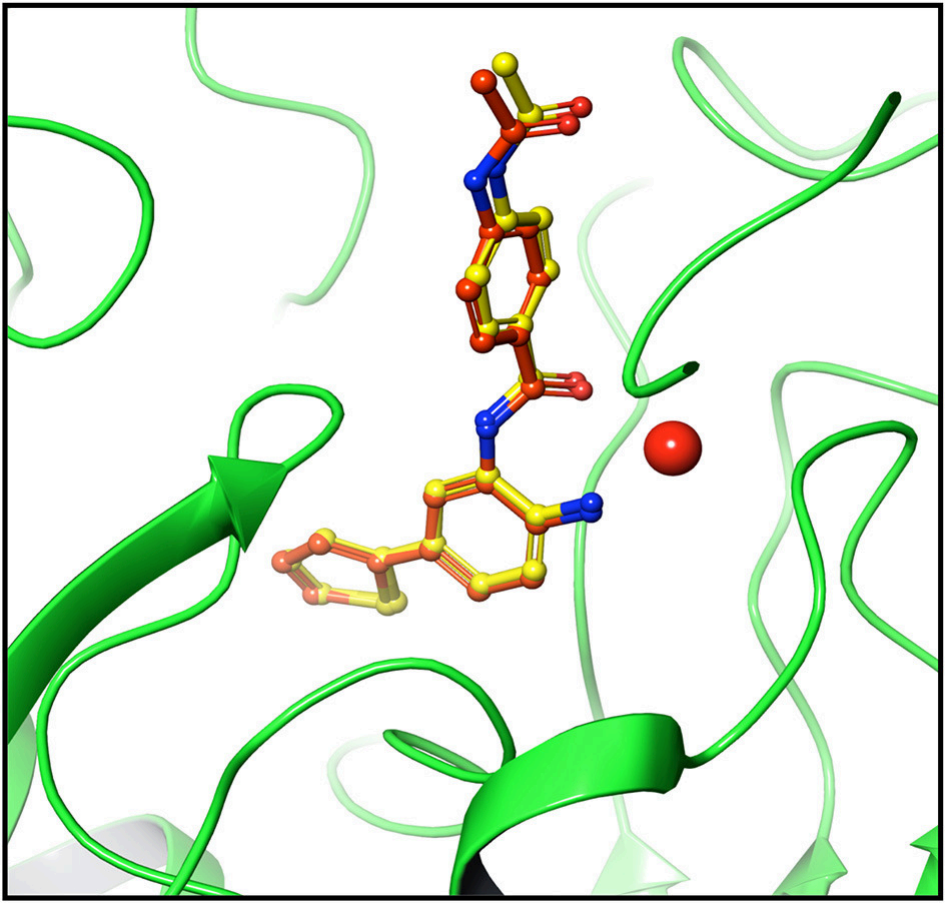
Pose validation for testing docking algorithm. Protein was minimized along with native ligand. Ligand was separated and protein was subjected to grid generation. The separated native ligand was redocked with the host protein using extra precision flexible docking method and root mean square deviation (RMSD) was calculated between native pose (yellow) and redocked pose (dark brown). The native and redocked pose showed RMSD value of 0.39 Å thereby validating the ability of algorithm to determine active site and pose of ligand correctly. Cherry red sphere represents Zinc present at the active site.

For testing algorithm and selecting docking method pose validation was performed. Self-docking involving the docking of ligands into their native binding site provides a reasonable setup for evaluating docking programs and scoring functions (Sandor et al., 2010). The flexible docking protocol in extra precision mode reproduced a pose very close to crystal pose of ligand. The RMSD value of crystal and redocked pose was found to be 0.39 (Figure 2), authenticating the algorithm for reproducing correct pose (Figure 2). Thus, all the docking studies were performed using extra-precision flexible docking protocol (Jain, 2008).

### Molecular docking and evaluation of GScore

**Figure 3:**
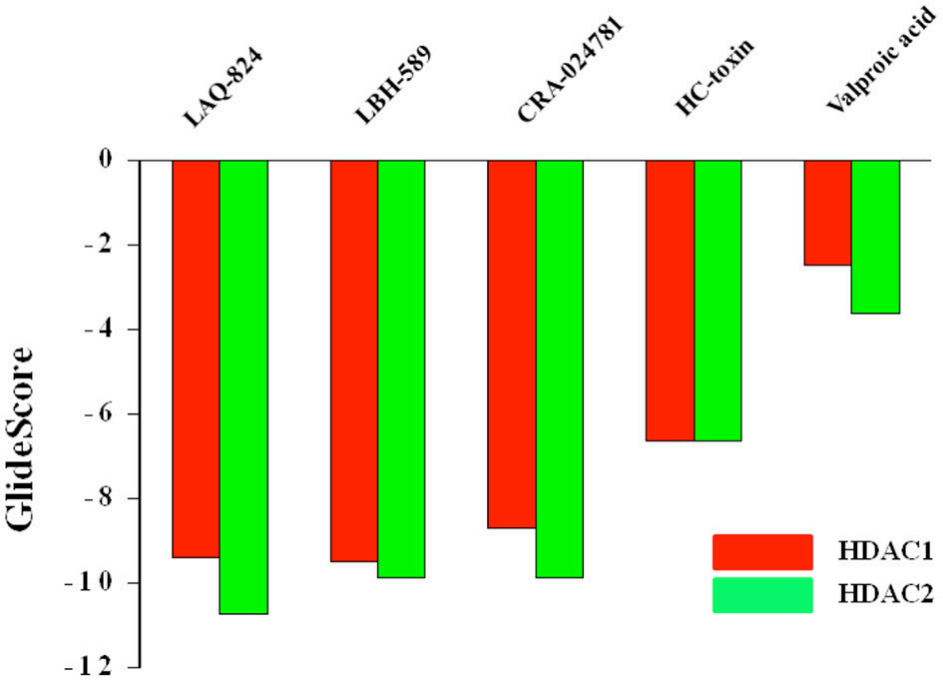
GScores of structurally distinct HDACi against HDAC1 and HDAC2 enzymes. More negative the GScore, more is the affinity of ligand toward receptor and vice versa.

Molecular docking, the central tool in drug discovery, provides details regarding the interaction profile of small molecules and a protein at atomic level. Studying such interactions sheds light on the behavior of small molecules in the binding pocket of target proteins. Moreover, these interactions play a considerable role in unraveling the principal biochemical processes (Meng et al., 2011). Accurate structural modeling and correct prediction of activity are regarded as the two chief aims of molecular docking (Ganai, 2016d). In order to gain insights on substantial ligand characteristics arising during the interaction between HDACs and their inhibitors, 5 HDACi from distinct structural groups were chosen (Supplementary Figure 1). These inhibitors with previously determined *in vitro* IC_50_ values under a similar experimental setup (Bradner et al., 2010) were docked against HDAC1 and HDAC2 isoforms. The GScore obtained from 10 docked complexes are shown in Figure 3 and Supplementary Table 1. More negative the GScore more is the affinity of ligand toward receptor and vice versa. Among the chosen HDACi, LAQ-824 and other hydroxamates showed most favorable (more negative) GScore. These results are in consistent with the previous reports where hydroxamates and benzamide derivatives showed highest GScore against Class II HDACs and short chain fatty acids like sodium butyrate showed least negative GScore (Ganai et al., 2015b). Similar results were reported by another group against class I HDACs. LAQ824 proved to be the first highest scoring among hydroxamates showing a GScore of −10.74 against HDAC2. While HC-toxin, the cyclic peptide group inhibitor showed GScore of −6.64, valproic acid, the short chain fatty acid group inhibitor showed GScore of −3.61 against HDAC2.

However, unlike HDAC2 the first most favorable inhibitor in terms of GScore proved to be LBH-589 (−9.51). This was followed by LAQ824 as evident from a GScore of −9.4. While HC-toxin displayed a GScore of −6.65, valproic acid proved to be feeble inhibitor against HDAC1 as evidenced by least negative GScore (−2.48) respectively. These finding correlate well with the previous findings reporting short chain fatty acids like butyrate and valproic acid as feeble inhibitors of HDACs (Dokmanovic et al., 2007; Rasheed et al., 2007).

Our docking studies revealed that among hydroxamates LAQ824 has strong binding potential especially against the HDAC2 and valproic acid has feeble affinity toward both HDACs. Moreover, HC-toxin, a cyclic peptides showed considerable affinity score against HDAC1 and HDAC2 enzymes. Thus, in the future experiments we tried to explore the reason of differential affinity of hydroxamates, cyclic peptides and short chain fatty acid valproic acid against these Class I isoforms.

### Ability to form multiple interactions markedly enhances affinity score

**Figure 4:**
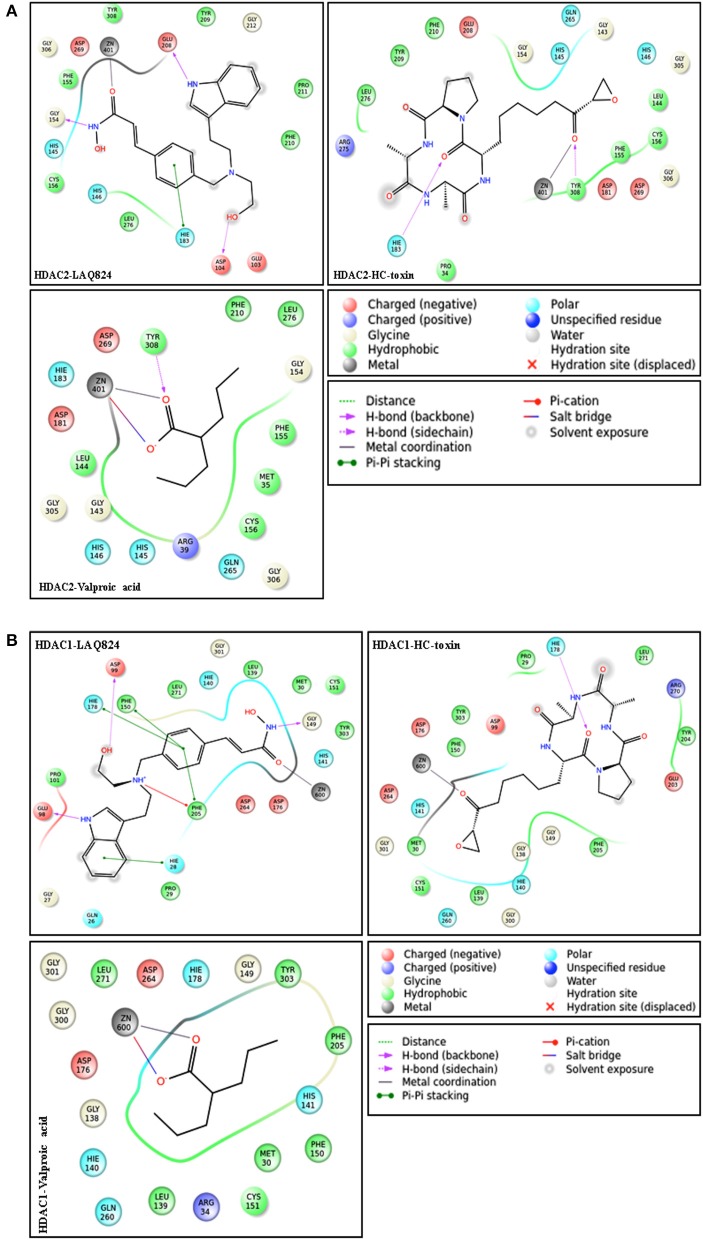
**(A)** Ligand interaction diagrams of structurally different HDACi against HDAC2. Diagrams were generated from poses having highest negative GScore using default parameters (upto 4 Å distance from ligand). LAQ824 forms more interactions with the receptor compared to valproic acid and thus shows more negative GScore. Kindly refer lid for more explanation. **(B)** Ligand interaction profile of different HDACi against HDAC1.

In order to gain insights about the mechanistic details resulting in the strong affinity score of hydroxamates and the weakest affinity of short chain fatty acid group HDACi, ligand interaction profile of 10 docked complexes were generated. This profile highlights the different interactions possible between ligand and receptor. Hydroxamates like LAQ-824 and LBH589 displayed various interactions with the receptors HDAC2 and HDAC1. These inhibitors only showed highest negative GScore (strong affinity score) during molecular docking studies. Short chain fatty acids, such as phenylbutyrate and valproic acid form only a few interactions with the defined receptors. These inhibitors showed least negative GScore (weak affinity) especially in case of HDAC1. LAQ824, the highest scoring hydroxamate showed three hydrogen bond interactions, one Pi-Pi stacking with HDAC2. While the hydrogen bonding residues with the aforementioned inhibitor were Glu 208, Asp 104 and Gly 154, His 183 was found to be involved in Pi-Pi stacking. On the other hand valproic acid, the short chain fatty acid formed one hydrogen bond with Tyr 308, one salt bridge and metal coordination with active site Zinc ion of HDAC2 (Figure 4A). Similarly hydroxamates LAQ-824 interact with HDAC1 through various bondings in contrast to short chain fatty acid valproate. LAQ-824 interacts via three hydrogen bonds (Gly149, Asp199, and Glu98), four Pi-Pi stacking (Phe205, Phe150, His178, His28) and one Pi-cation interaction (Phe205) with the active site of HDAC1 (Figure 4B). While hydroxamate CRA024781 form salt bridges (Arg39 and Glu103 respectively) with the binding pocket of HDAC2, no such interactions were seen in case of HDAC1 except in case of valproic acid which forms salt bridge with Zinc cation. The only cyclic peptide inhibitor HC-toxin forms two and one hydrogen bonds with the active site residues of HDAC2 (His183 and Tyr308) and HDAC1 (His178) respectively.

Speaking in general, the high affinity of hydroxamate HDACi may be attributed to higher number of aromatic rings present in these inhibitors. The presence of aromatic rings in these inhibitors results in Pi-Pi stacking and Pi-cation interactions, making their GScore more negative. Moreover, the hydroxamate group in these inhibitors forms hydrogen bonds with HDAC1 and HDAC2 active site besides forming metal coordination with Zinc. Short chain fatty acid valproic acid devoid of any aromatic ring lacks the ability to form Pi-Pi stacking and Pi-cation interactions, which reduces its negative GScore drastically. Taken together, molecular docking studies and ligand interaction profile analysis shows hydroxamates as potent inhibitors against HDAC1 and HDAC2. Moreover, HC-toxin, a cyclic peptide showed better interaction profile against HDAC1 and HDAC2 enzymes. Thus, in nutshell, hydroxamates and cyclic peptide HC-toxin show better interaction profile with these enzymes. In order to confirm these findings we used molecular mechanics generalized born surface area (MMGBSA), an implicit solvation model to calculate the binding free energy of ligand-receptor complexes.

### Binding free energy values aligned well with *in Vitro* IC_50_ values

**Figure 5:**
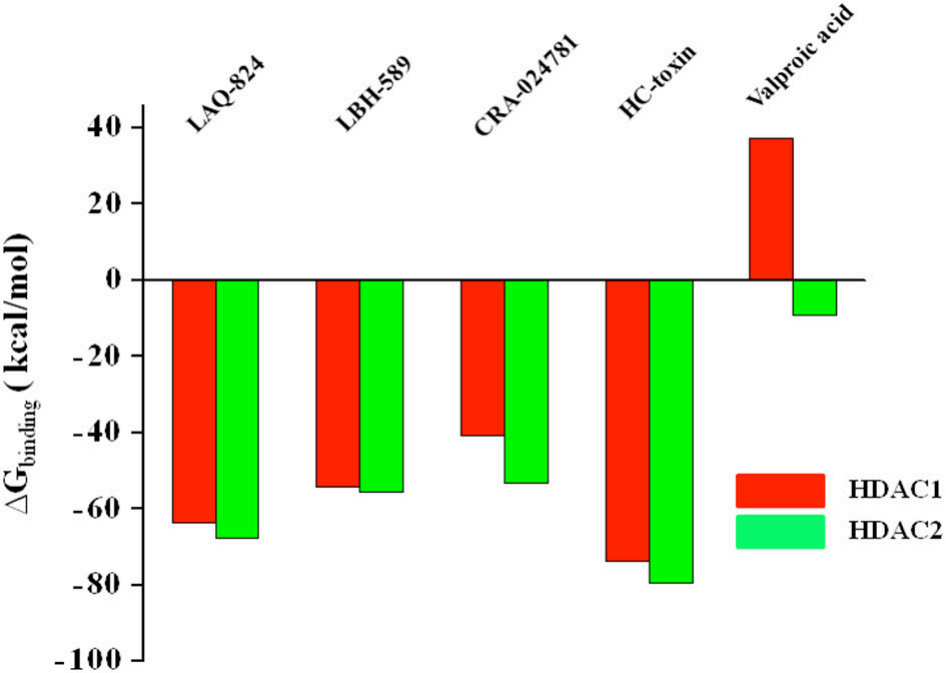
BFE values of structurally different HDACi against HDAC1 and HDAC enzymes in frozen state where no flexibility was given to receptor. The binding free energy values reflect the affinity of ligand toward receptor. More negative the value, more is the affinity and vice versa.

The binding free energy of 10 docked complexes was calculated by using MM-GBSA under default conditions (Singh and Muthusamy, 2013). The calculated values showed parallel trend with the *in vitro* IC_50_ values of the respective inhibitors (Bradner et al., 2010). More negative the value of binding free energy more is the affinity between ligand and receptor and vice versa. Similarly lower the IC_50_, more potent is the inhibitor and thus IC_50_ is inversely proportional to potency of inhibitor (Ganai et al., 2015b). Hydroxamate LAQ824 with an IC_50_ of 0.003 μM against HDAC2 showed binding free energy (BFE) value of −67.7911 kcal/mol. HC-toxin, a cyclic peptide (IC_50_ = 0.9 μM) displayed a BFE value of −79.5371 kcal/mol while a value of only −9.09224 kcal/mol was obtained for short chain fatty acid valproic acid (IC_50_ of 75 μM) against the defined HDAC (Figure 5 and Supplementary Table 2). For HDAC1, LAQ824, a hydroxamate (IC_50_ 0.001 μM) showed a BFE value of −63.61 kcal/mol while valproic acid with higher value of 51 μM showed lesser negative value of binding free energy (37.09804 kcal/mol) (Bradner et al., 2010). Thus, it is quite evident that hydroxamates having lower IC_50_ values against HDAC1 and HDAC2 show more negative values of binding free energy while short chain fatty acid valproic acid having higher IC_50_ value showed very poor value expectedly. The parallel trend between binding free energies and the *in vitro* IC_50_ values especially in case of hydroxamates and short chain fatty acid was obtained using the default frozen state where no flexibility was given to receptors HDAC1 or HDAC2. These results are in consistent with the previous findings where maximum correlation has been seen reported for β2-adrenergic receptor agonists and antagonists when the receptor was held frozen (Vilar et al., 2010).

Thus calculation of binding free energy using MM-GBSA strongly supported our molecular docking predictions. On analyzing all the three parameters including GScore, interaction profile and binding BFE values we confined our downstream experiments to top scoring hydroxamate LAQ824 and the only cyclic peptide HC-toxin and ignored short chain fatty acid valproic acid due to its poor interaction profile and BFE. The main aim of our study was to generate e-pharmacophores against HDAC1 and HDAC2 enzymes for isoform selective inhibition. Generating these pharmacophores directly from docked complexes does not address the ligand stability and hence the energy issue. Thus, we performed MDS prior to creation of e-Pharmacophores for docked complexes to overcome this impediment.

### Molecular dynamics simulation confirmed receptor stability throughout simulation

**Figure 6:**
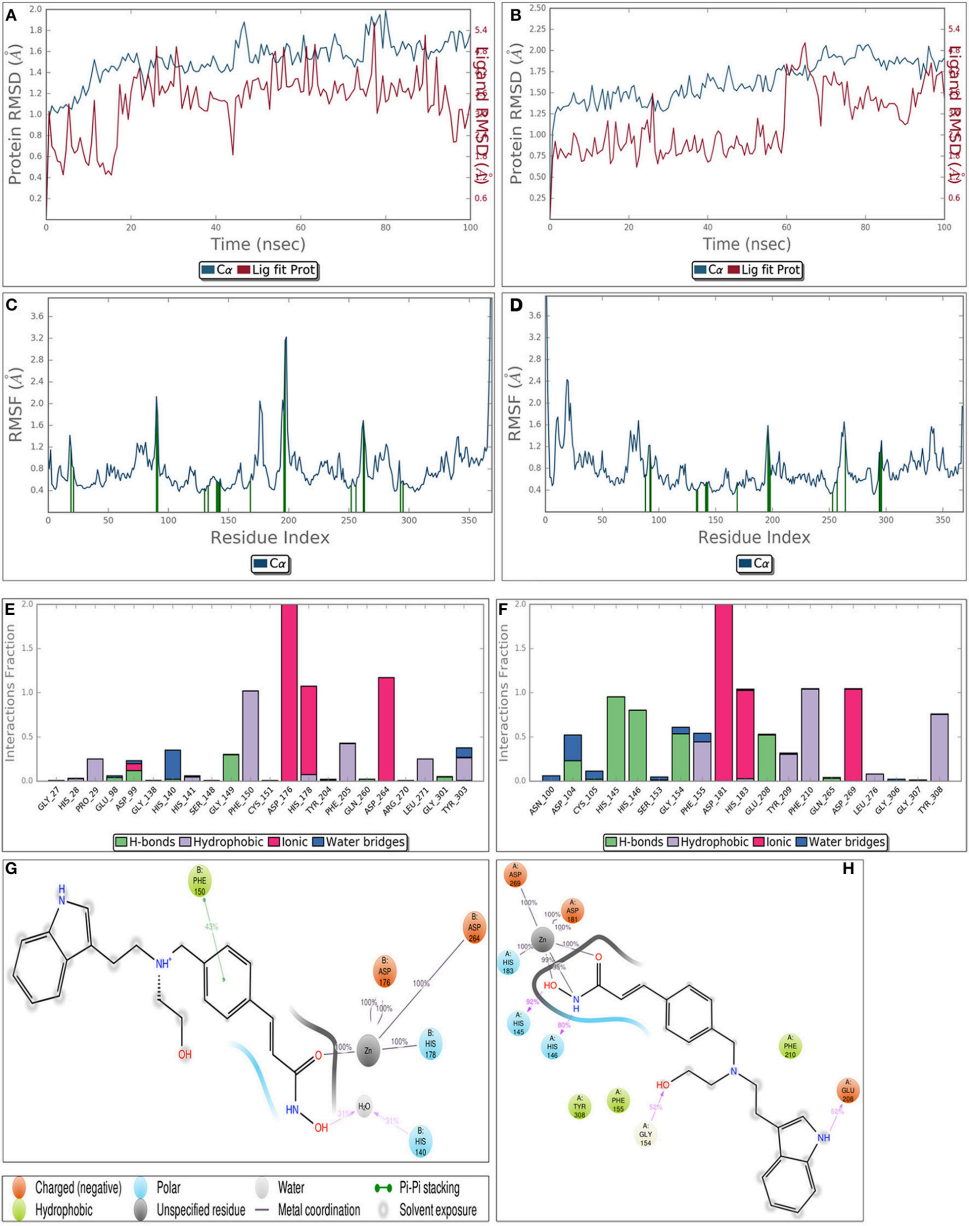
Molecular dynamics simulation of LAQ824-HDAC1 **(A)** and LAQ824-HDAC2 **(B)** docked complexes around 100 ns. RMSD is calculated for all frames and for frame x is:
$$RMSD_{X}=\sqrt{\frac{1}{N}\sum_{i=1}^{N}{\left(r_{i}^{\prime }\left(t_{x})\right)-r_{i}\left(t_{ref}\right)\right)}^{2}}$$
Here N represents number of atoms in atom selection; *t*_*ref*_ designates reference time, first frame is selected as reference and is considered as time *t* = 0; *r*′ represents the position of selected atoms in frame x after aligning on the reference frame, where frame x is recorded at time *t*_*x*_. The RMSD of proteins (blue line) is well below 3 clearly suggesting the protein is pretty stable throughout simulation. Moreover, the results show that LAQ824 is quite stable in the active site of these proteins. **(C,D)** represent the Root Mean Square Fluctuation (RMSF) for LAQ824-HDAC1 and LAQ824-HDAC2. It has importance in characterizing local changes along the protein chain. RMSF for residue *i* is calculated as:
$$RMSF_{i}=\sqrt{\frac{1}{T}\sum_{i=1}^{T}<{\left(r_{i}^{\prime }\left(t)\right)-r_{i}\left(t_{ref}\right)\right)}^{2}>}$$
In this equation, *T* signifies trajectory time over which RMSF is calculated, *t*_*ref*_ denotes reference time; *r*_*i*_ represents position of residue *i*; *r*′ designates the position of atoms in residue i after superposition on the reference, average of square distance is taken over the selection of atoms in residue is indicated by angle brackets. Peaks represent the areas fluctuating maximum during the simulation. The N and C terminal regions relatively fluctuate more than any other protein part. The loop regions show more fluctuation than rigid protein regions including alpha helices and beta strands. Green colored vertical bars represent the protein residues interacting with ligand (LAQ824). Different types of interactions between LAQ-824-HDAC1 **(E,G)** and LAQ824-HDAC2 **(F,G)** throughout the simulation. A value of 1.0 in stacked bar charts represents that during 100% of the simulation time a particular interaction is maintained. Ligand interaction diagrams represent interactions sustaining over 30% of the simulation time. Refer lid given on side for various bonds. It is quite evident that HDACi form metal coordination with Zinc ion present at the active site of HDACs and thus disrupt charge relay mechanism.

**Figure 7:**
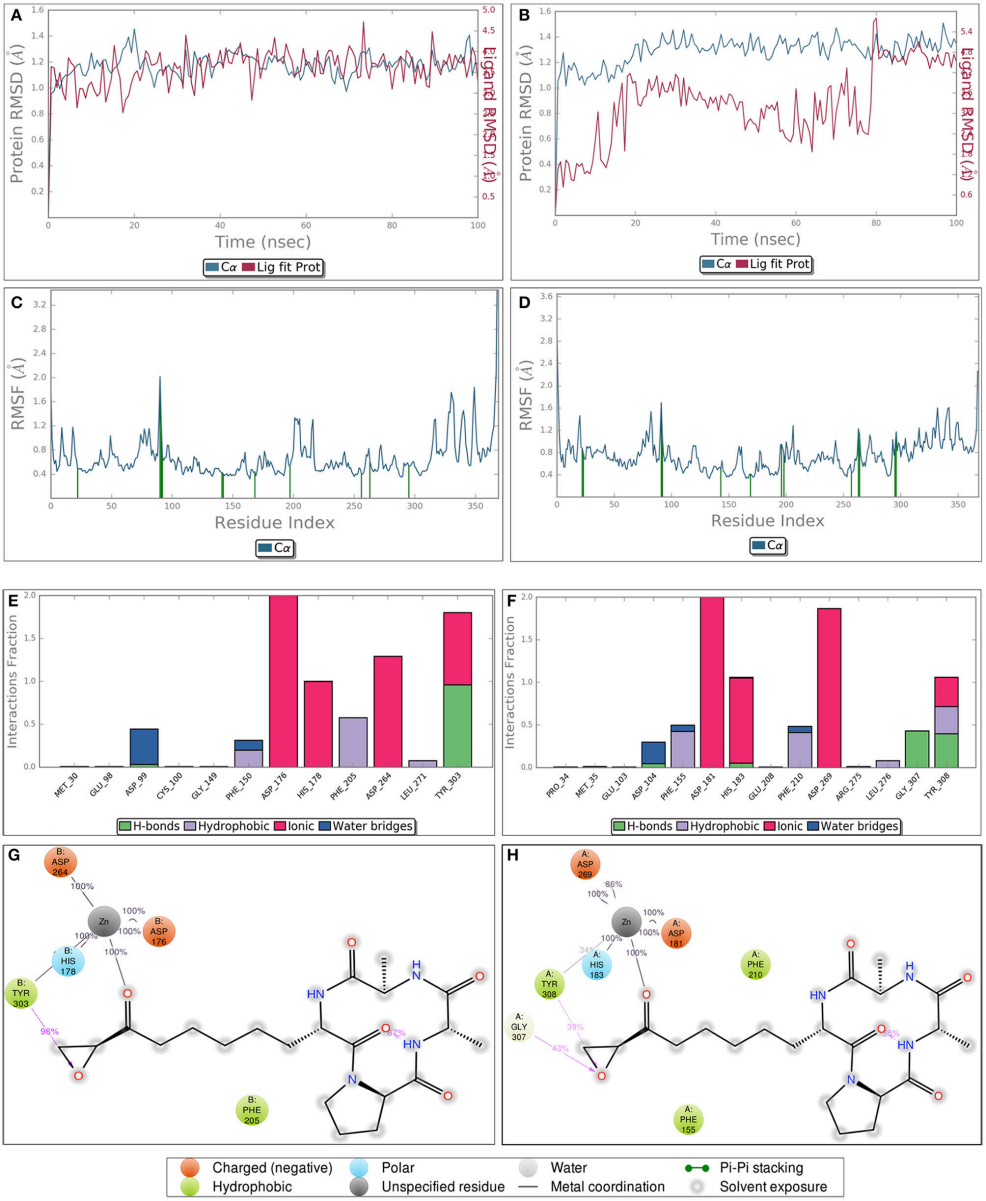
Diagrams obtained after performing molecular dynamics simulation of HC-toxin-HDAC1 and HC-toxin-HDAC2 docked complexes for around 100 ns. While **(A,B)** indicate the RMSF of HDAC1 and HDAC2 in docked state with HC-toxin, **(C,D)** represent the RMSF of the defined HDACs in bound state with the HC-toxin. Different types of interactions between HC-toxin and the two HDACs. **(E,G)** represent interaction profile of HC-toxin-HDAC1; **(F,H)** represent same profile for HC-toxin-HDAC2. Consider interactions made by HC-toxin with receptor which includes both HDAC1 and HDAC2 and their cofactor Zn.

Though static structure-based approaches like molecular docking and virtual screening have contributed significantly to advanced drug discovery, but they do not take into account, the dynamic nature of proteins (Gunasekaran and Nussinov, 2007). As our studies are related to inhibitor induced conformational changes of active sites where this dynamic nature cannot be breached (Gutteridge and Thornton, 2005; Samsonov et al., 2014), we coupled MDS to these static approaches to overcome the defined loophole. MDS provides in depth insights about small-molecule receptor stability and compatibility and consequently the competency of these molecules to modulate receptor physiological function. Taking these grim facts into consideration, we performed MDS for 100 ns for the most potent inhibitors LAQ824 and HC-toxin in docked state with HDAC1 and HDAC2. RMSD of protein was calculated by aligning all protein frames on the reference frame backbone. Monitoring the RMSD provides crucial details about the structural conformation of protein all through the simulation. Our MDS studies showed protein RMSD well below 3 Å clearly suggesting that docked complexes HDAC1-LAQ824, HDAC2-LAQ824, HDAC1-HC-toxin, and HDAC2-HC-toxin are highly compatible with each other (Figures 6A–D). The RMSF is beneficial for delineating local changes along the protein chain. The secondary structure elements like alpha helices and beta strands portrayed less fluctuation compared to loop regions expectedly (Figures 6A–D). While in case of HDAC1, the residues critical for interaction with LAQ824 are Phe 150, Phe 205, Tyr 303, His 140, Gly 149, Pro 29, and Asp 99 the residues Phe 210, His 145, His 146, Tyr 308 Gly 154, Phe 155, Glu 208, and Asp 104 are crucial in case of HDAC2 (Figures 6E,F). HC-toxin interacts with Tyr 303, Phe 205, Asp 99 and Phe 150 residues of HDAC1 (Figures 6G,H). Regarding HDAC2, HC-toxin portrayed interactions with Tyr 308, Phe 210, Phe 155, Gly 307, and Asp 104 (Figures 7A–H). Previous studies have shown that classical HDACs deacetylate histone substrates using a charge relay mechanism like serine proteases. In this mechanism histidine and aspartate residues participate in addition to Zinc ion (Finnin et al., 1999). Our current findings established that HDACi chelate Zinc ion and thus inactivate classical HDACs (Figure 5). These findings are in consistent with the earlier findings where HDACi have been reported to disrupt charge relay mechanism in case of class II HDACs through Zinc chelation (Ganai et al., 2015b).

Speaking concisely our molecular dynamics studies showed that HDAC1 and HDAC2 are quite stable throughout simulation. Moreover, in the protein-ligand contact diagrams it is quite evident that HDACi target Zinc ion, one of the crucial player in charge relay mechanism. The main aim in this study was to develop e-Pharmacophores from the highly stable pose of ligand which can be obtained only by serious analysis of MDS trajectory. Thus, we performed clustering of MDS output file using Desmond trajectory clustering prior to e-pharmacophore approach.

### Creating pharmacophoric features of top scoring inhibitors against HDAC1 and HDAC2 enzymes

Pharmacophore according to Paul Ehrlich is a molecular framework responsible for the biological activity of the drug. According to IUPAC, pharmacophore is an ensemble of steric and electronic features vital for ensuring the optimal supramolecular interactions with a particular biological target and to activate or impede its biological response. The information regarding the common properties among the different binding groups is indispensable for determining the type of inhibitors that are binding the receptor. The pharmacophoric predictions provide such information and thus augment the guidance for the rational design of novel molecules (Zhu et al., 2010). Taking these facts into consideration, we explored the pharmacophoric features of HDACi belonging to structurally distinct groups against HDAC1 and HDAC2 isoforms. In ligand based pharmacophore modeling receptor is not known and the common hypothesis is generated from the active ligands against that receptor (Vuorinen et al., 2014). The energy based models developed in the present work involves the docking of bioactive small-molecule into the active site of receptor. The e-Pharmacophore approach uses the Glide XP scoring function to precisely characterize protein-ligand interactions (Loving et al., 2009; Salam et al., 2009; Ganai, 2016d). As this approach also involves receptor to generate complimentary features, one active compound is sufficient to achieve logical conclusion (Ganai et al., 2015b). As aforementioned, the previous studies have been generated e-pharmacophores directly from docked complexes as thus do not take into account the ligand stability, we used a novel strategy of coupling molecular dynamics to e-pharmacophore via Desmond trajectory clustering, a script which performs hierarchical clustering using MDS output file of protein-ligand complex as input. The MDS trajectory of each docked complex was separated into 7 clusters and the one with maximum number of frames was used for generating e-Pharmacophores. We used auto e-pharmacophore of Phase for generating the critical features of LAQ824, a hydroxamate representative and HC-toxin, a cyclic tetrapeptide against HDAC1 and HDAC2, class I isoforms.

#### LAQ824 showed differential features against HDAC1 and HDAC2 enzymes

**Figure 8:**
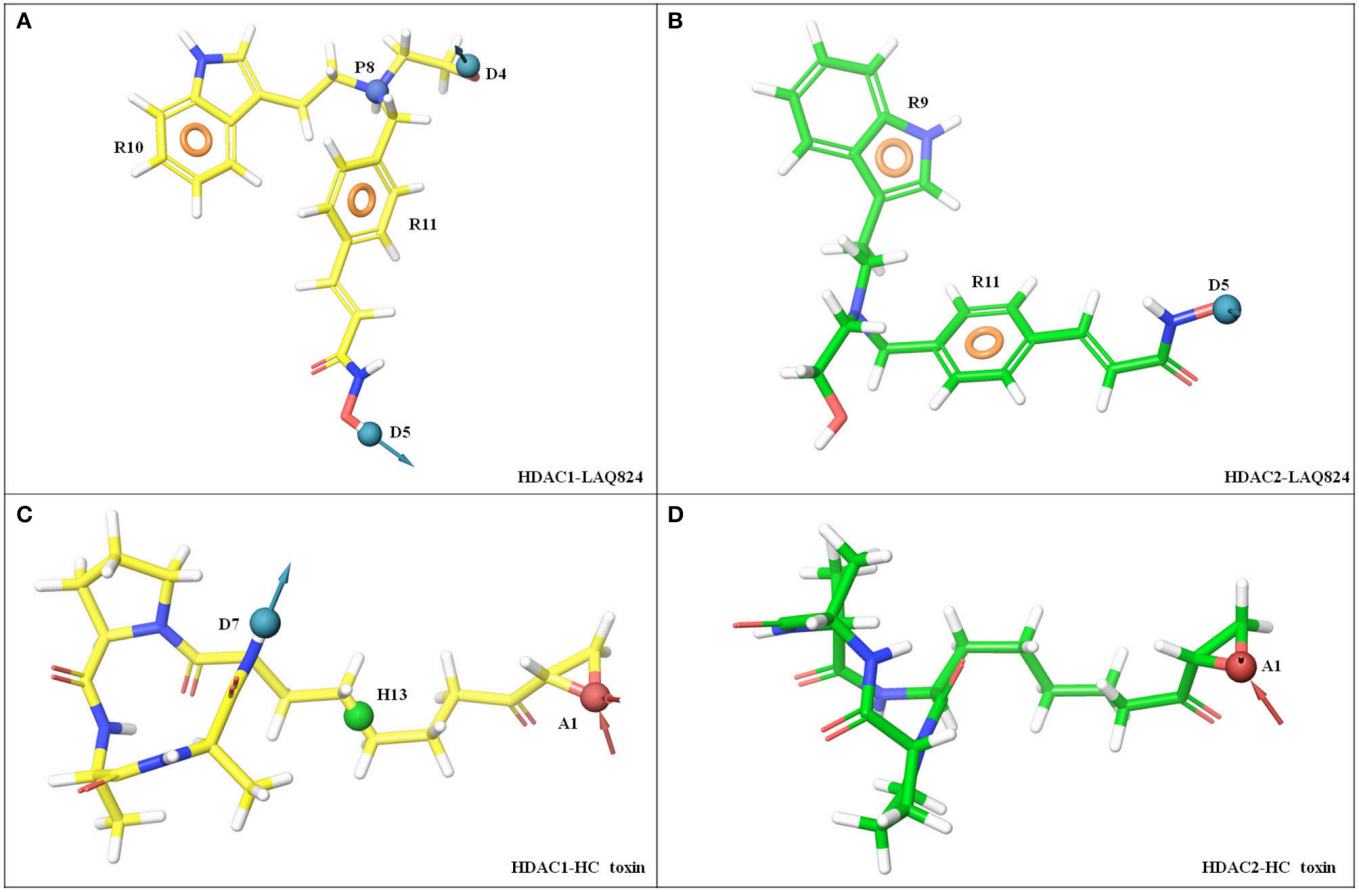
E-Pharmacophoric features of LAQ824 and HC-toxin against HDAC1 and HDAC2 enzymes. LAQ824 shows distinct features against HDAC1 and HDAC2. For HDAC1, LAQ824 portrays five features while for HDAC2 it shows only three features. Two extra features both in linker region of LAQ824 (P8 and D4) were seen for LAQ824 while in the binding pocket of HDAC1 as compared to HDAC2. While HC-toxin portrayed three features (hydrophobic group in linker region, HBA in ZBG and HBD in cap region) against HDAC1, only one feature (HBA in ZBG) was seen against HDAC2. **(A)** HDAC1- LAQ824, **(B)** HDAC2- LAQ824, **(C)** HDAC1- HC-toxin, and **(D)** HDAC2- HC-toxin.

As aforementioned the pharmacophores were generated from cluster with highest frame frequency. LAQ824 portrayed distinct features against HDAC1 and HDAC2. The defined inhibitor showed five features against HDAC1 namely two hydrogen bond donors (D5) in ZBG and D4 in linker region; two aromatic rings one in linker (R11) and another in cap region (R10); one positive ionizable group (P8) in linker region. Contrary to HDAC1, LAQ824 showed three features against HDAC2 namely one HBD's (D5) in ZBG; two aromatic rings (R9 and R11) in cap and linker region respectively. Thus, LAQ824 showed two features (P8 and D4) lesser against HDAC2 in comparison to HDAC1 (Figures 8A,B).

### 2 HC-toxin showed two features less for inhibiting HDAC2 compared to HDAC1

HC-toxin showed three distinct features namely hydrogen bond acceptor (HBA) in ZBG (A1); one hydrophobic group (H13) in linker region and one hydrogen bond donor (HBD) in cap region. For HDAC2, it portrayed only one HBA (A1) in ZBG (Figures 8C,D). Thus, the above findings suggest that for HDAC2 specific inhibition, LAQ824 pharmacophore should have one HBD and one positive ionizable group (both in linker region) less for HDAC2 as compared to HDAC1. While for type-specific inhibition of HDAC1, HC-toxin pharmacophore should have three features located in ZBG, linker and cap, only single feature HBA in ZBG is required for HDAC2 isoform.

#### LAQ-824 and HC-toxin showed differential high energy features against HDAC1 and HDAC2

Energy contribution of each pharmacophoric feature was calculated for LAQ824 and HC-toxin against HDAC1 and HDAC2 enzymes. While positive ionizable group (P8) in linker region (R_L_) was maximum scoring (−4.07 kcal/mol) against HDAC1, aromatic ring (R11) in linker (R_L_) proved to be best scoring feature (−1.26 kcal/mol) in case of HDAC2. Regarding HC-toxin, HBA in ZBG (A_Z_) scored maximum in terms of energy (−0.7 kcal/mol) against HDAC1, followed by hydrophobic group in linker and HBD in cap. However, for HDAC2, HBA in ZBG (A_Z_) was the sole top scoring feature (−0.7 kcal/mol). The details of various pharmacophoric features with rank wise scores are summed in Table 1. Summarizing in few words P_L_ > D_Z_ > R_L_ > R_C_ > D_L_ in case of LAQ824-HDAC1 while R_L_ > D_Z_ > R_C_ in case of LAQ824-HDAC2 (Table 2). From these studies it is clear that a single inhibitor exhibits differential features against HDAC1 and HDAC2 isoforms and thus in the downstream steps we used the e-Pharmacophores of HDAC1-LAQ824 and HDAC2-LAQ824 as queries in e-Pharmacophore based virtual screening and selected five hits for each of the e-Pharmacophores and tested them against the respective receptors for inhibition.

**Table 1:** Ligand interaction profile of HDACi against HDAC1 and HDAC2 enzymes.

**HDAC inhibitor group**	**HDAC inhibitor**	**HDAC2**			**HDAC1**			
**Hydroxamates**		**HBR**	**Pi-Pi stacking**	**Salt bridge**	**HBR**	**Pi-Pi stacking**	**Pi-cation**	**Salt bridge**
	LAQ-824	ASP104	HIS183		GLY149	PHE205	PHE205	
		GLU208			ASP99	PHE150		
		GLY154			GLU98	HIS178		
						HIS28		
	CRA-024781	ASP104	PHE155	GLU103	GLY149	HIS141		
		GLY154			HIS178			
	LBH-589	ASP104	HIS33		GLY149	PHE150	PHE205	
		GLU103	HIS183		GLU98	HIS178		
		GLY154	PHE210		ASP99			
Cyclic peptides	HC-toxin	HIS183			HIS178			
		TYR308						
Short chain fatty acids	Valproic acid	TYR308		Zn401				Zn600

**Table 2:** Energy contribution of individual pharmacophoric features against HDAC1 and HDAC2, class I isoforms.

**HDAC inhibitor**	**Target HDAC**	**Feature label**	**Component location**	**Score (Rank wise)**
LAQ824	HDAC1	P8	P_L_	−4.07
		D5	D_Z_	−1.35
		R11	R_L_	−0.89
		R10	R_C_	−0.67
		D4	D_L_	−0.34
	HDAC2	R11	R_L_	−1.26
		D5	D_Z_	−1.22
		R9	R_C_	−0.8
HC-toxin	HDAC1	A1	A_Z_	−0.7
		H13	H_L_	−0.34
		D7	D_C_	−0.17
	HDAC2	A1	A_Z_	−0.7

*High scoring features of representative HDACi against HDAC1 and HDAC2 enzymes. AL, AC, AZ designate HBA in linker, cap and ZBG. While DL, DC, and DZ signify HBD in linker region, cap region and ZBG respectively, RL, RC, RZ represent ring in the predefined regions. Last but no way least HL represents hydrophobic group in linker region of inhibitor. PL designates positive ionizable group in linker region from the table it is quite evident that maximum energy contributing feature in LAQ824 against HDAC1 is PL while it is RL in case of HDAC2. In case of HC-toxin, AZ scores maximum against both HDAC1 and HDAC2 in case of HC-toxin*.

### Hits retrieved from e-Pharmacophores based virtual screening portrayed promising affinity toward therapeutically relevant HDAC2

**Figure 9:**
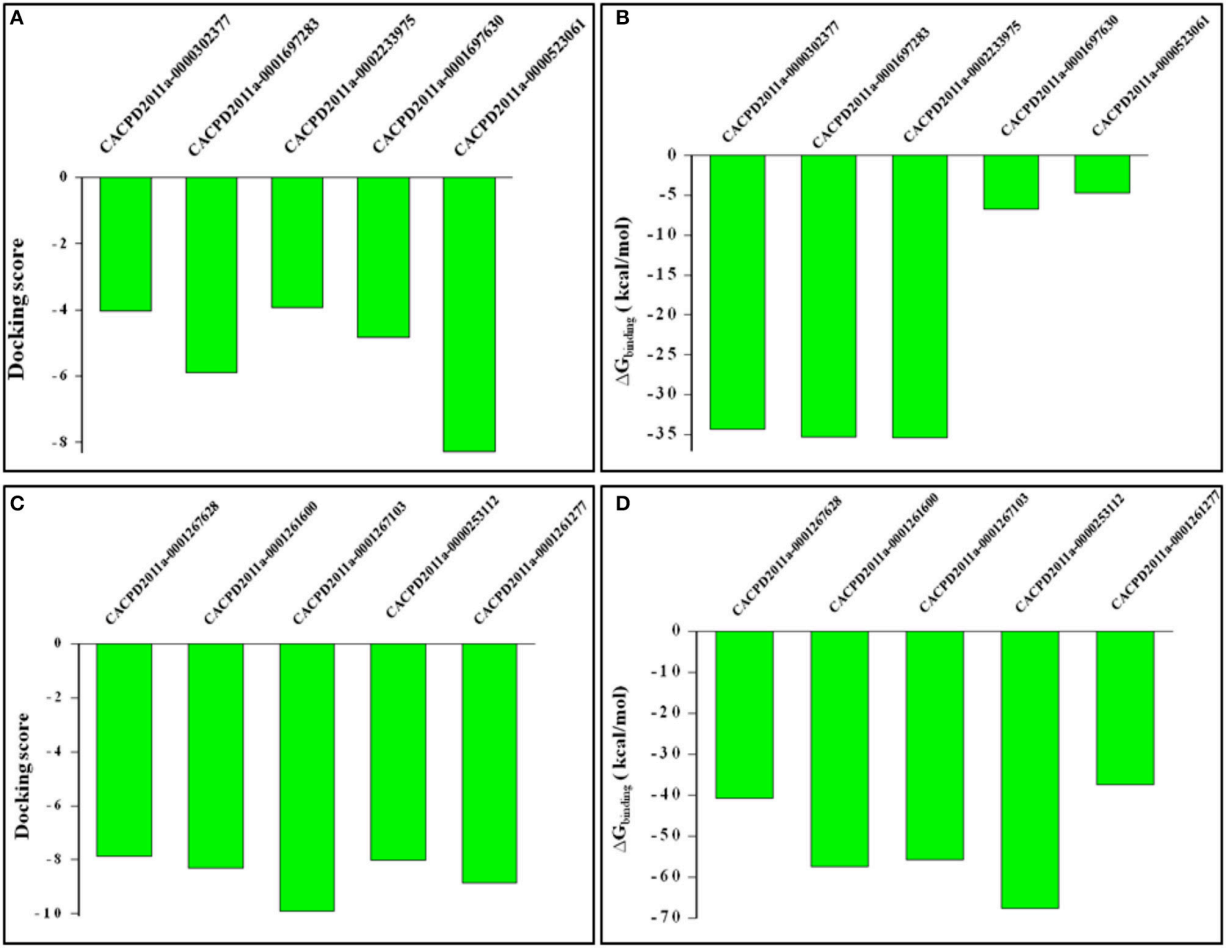
Docking scores and binding free energy values of virtually screened hits against HDAC1 and HDAC2. While **(A,B)** shows docking scores and binding free energy values of hits against HDAC1, **(C,D)** designate the defined parameters for HDAC2.

The hit molecules retrieved from e-Pharmacophores based virtual screening were selected based on fitness and logic as described in methodology section. For HDAC1, five hits having CACPD2011aCode CACPD2011a-0000302377, CACPD2011a-0001697283, CACPD2011a-0002233975, CACPD2011a-0001697630, and CACPD2011a-0000523061 (Supplementary Figure 2) were finally selected among more than 1000 hits. For HDAC2, CACPD2011a-0001267628, CACPD2011a-0001261600, CACPD2011a-0001267103, CACPD2011a-0000253112, and CACPD2011a-0001261277 (Supplementary Figure 3) were selected among the retrieved hits. The selected hits obtained from HDAC1-LAQ824 and HDAC2-LAQ824 were docked against HDAC1, and HDAC2 respectively. These hits showed negative values of docking score and negative values of binding free energy clearly indicating that these hits do inhibit the respective isoforms and thus validating the e-Pharmacophores hypothesis. Among the selected hits for HDAC1, the first three compounds showed most favorable (more negative) docking as well as BFE values (Figures 9A,B). Regarding the therapeutically relevant HDAC2, all the five hits in general and the hits second, third and fourth in particular portrayed promising affinity toward HDAC2 as evidenced by the expectedly more negative values of docking score and BFE (Figures 9C,D).

## Conclusion

In this work we have taken the advantage of combinatorial *in silico* approach including XP-molecular docking, MMGBSA, MDS, trajectory clustering and e-Pharmacophores approach and e-Pharmacophores based virtual screening to exploit the significances of various structural variants in the HDAC inhibitor-HDAC1 and HDAC inhibitor-HDAC2 complexes. HDACi from three distinct structural groups; hydroxamates, cyclic tetrapeptides and short chain fatty acids were docked against HDAC1 and HDAC2 enzymes for evaluating the interaction mechanisms and affinity scores. The predicted BFE values aligned well with the *in vitro* IC_50_ values of inhibitors thereby validating the simulation accuracy. Our molecular docking studies demonstrated that hydroxamates like LAQ824, LBH589 etc., and cyclic tetrapeptide HC-toxin display higher affinities with structurally identical HDAC1 and HDAC2, class I isoforms. Moreover, these studies confirmed that inhibitors with higher number of aromatic rings like LAQ824 show enhanced potency while lacking such rings (valproic acid) show feeble affinity toward these enzymes.

Classical HDACs deacetylate histone substrates using charge relay mechanism in which two histidine, two aspartate residues and Zinc ion play a crucial role. Our MDS studies showed that HDACi chelate the Zinc ion by interacting with it thereby disrupting charge relay mechanism, providing further support to the previous findings of Finnin et al. (1999) and Ganai et al. (2015b). We performed MDS of docked complexes followed by MDS trajectory clustering for confirming the stability of protein throughout simulation and for understanding the ligand stability. Our e-Pharmacophores approach proved that HDACi LAQ824 and HC-toxin show differential features against the structurally identical HDAC1 and HDAC2, Class I isoforms. Presence of positive ionizable group and HBD in the linker region of LAQ-824 has significance for inhibiting HDAC1 while absence of these two features is meaningful in case of HDAC2. These findings suggest that presence of positive ionizable group and HBD in linker region of LAQ-824 favors its selectivity toward HDAC1 while absence of these features shifts the selectivity toward HDAC2. The presence of HBD in cap region and hydrophobic group in linker region of HC-toxin promotes its selectivity toward HDAC1 while deficiency of these features enhances its selectivity toward HDAC2.

Quantitative analysis of energy contribution of each pharmacophoric feature was performed for LAQ824-HDAC1, LAQ824-HDAC2, HC-toxin-HDAC1, and HC-toxin-HDAC2 docked complexes. Our studies identified that the positive ionizable group in linker region (P_L_) of LAQ824 was the highest scoring pharmacophoric feature against HDAC1 whereas ring in linker (R_L_) scores maximum in the binding pocket of HDAC2. Hydrogen bond acceptor (HBA) in ZBG (A_Z_) was maximum scoring in case of both HDAC1 and HDAC2. Speaking concisely our *in silico* screening and e-Pharmacophore models tempt us to speculate that P_L_ and R_L_ of LAQ824 have a markedly significant role in inhibiting HDAC1 and HDAC2 enzymes respectively. From the above studies, it is quite evident that e-Pharmacophore models of same inhibitor shows different features against HDAC1 and HDAC2. The hits retrieved from e-Pharmacophores based virtual screening using the HDAC1-LAQ824 and HDAC2-LAQ824 pharmacophores as queries against phase database showed negative values of docking score and BFE thereby validating the accuracy of models. As the studies are focused on HDAC2, hits CACPD2011a-0001261600, CACPD2011a-0001267103, CACPD2011a-0000253112 proved to be most effective in inhibiting HDAC2. These hits with high potency and desired selectivity toward HDAC2 will play a key role in ameliorating neurodegenerative events safely while leaving the function of neuroprotective isoform intact.

## Author contributions

SG and MA designed research and wrote the paper. SG, EA, and RR performed experiments and analyzed data.

### Conflict of interest statement

The authors declare that the research was conducted in the absence of any commercial or financial relationships that could be construed as a potential conflict of interest.

## Supplementary material

Supplementary Figure 1 Name and structure of HDACi selected for study. The PubChem CID of HDACi is provided in bracket.

Supplementary Figure 2 Structures of five hits obtained through e-Pharmacophores based virtual screening for HDAC1. The 5th structure with CACPD2011aCode (CACPD2011a-0000302377) is the first hit, fourth structure (CACPD2011a-0001697283) is the second hit, third structure (CACPD2011a-0002233975) is the hird hit, structure second is the fourth hit and structure 1 (CACPD20111a-0000523061) is the 5th hit.

Supplementary Figure 3 Structures of hits selected for HDAC2 after virtual screening using LAQ824-HDAC2 e-Pharmacophores as query. Structure second (CACPD2011a-0001267628) is the first hit, structure first is the second hit, structure 5th is the third hit, structures third and fourth are the fourth and 5th hit respectively.
