# Supplementary figures and images for: Combinatorial In Silico Strategy towards Identifying Potential Hotspots during Inhibition of Structurally Identical HDAC1 and HDAC2 Enzymes for Effective Chemotherapy against Neurological Disorders
Source: Front Mol Neurosci. 2017 Nov 9;10:357. doi: 10.3389/fnmol.2017.00357 (PMC5684606; doi:10.3389/fnmol.2017.00357)

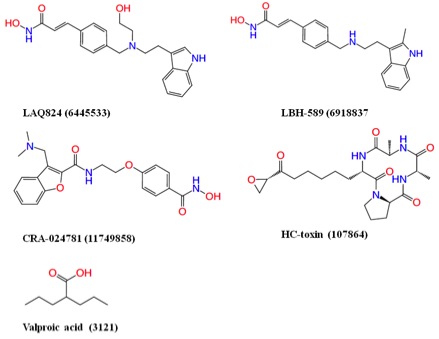

Supplement: Supplementary Figure 1 — Name and structure of HDACi selected for study. The PubChem CID of HDACi is provided in bracket. [file Image1.JPEG]

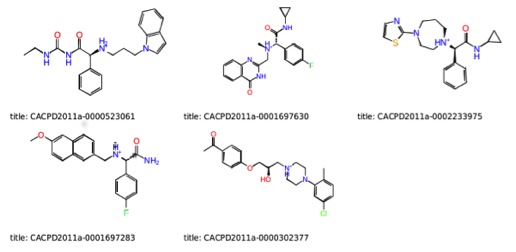

Supplement: Supplementary Figure 2 — Structures of five hits obtained through e-Pharmacophores based virtual screening for HDAC1. The 5th structure with CACPD2011aCode (CACPD2011a-0000302377) is the first hit, fourth structure (CACPD2011a-0001697283) is the second hit, third structure (CACPD2011a-0002233975) is the hird hit, structure second is the fourth hit and structure 1 (CACPD20111a-0000523061) is the 5th hit. [file Image2.JPEG]

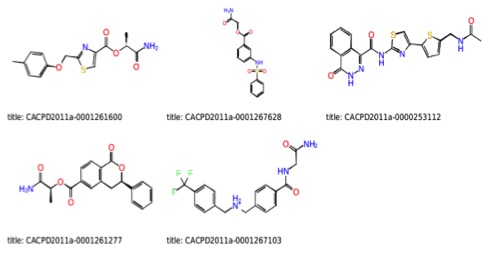

Supplement: Supplementary Figure 3 — Structures of hits selected for HDAC2 after virtual screening using LAQ824-HDAC2 e-Pharmacophores as query. Structure second (CACPD2011a-0001267628) is the first hit, structure first is the second hit, structure 5th is the third hit, structures third and fourth are the fourth and 5th hit respectively. [file Image3.JPEG]
