# Supplementary material for: Combinatorial In Silico Strategy towards Identifying Potential Hotspots during Inhibition of Structurally Identical HDAC1 and HDAC2 Enzymes for Effective Chemotherapy against Neurological Disorders
Source: Front Mol Neurosci. 2017 Nov 9;10:357. doi: 10.3389/fnmol.2017.00357 (PMC5684606; doi:10.3389/fnmol.2017.00357)
Supplement: Supplementary file 4 [file Table1.pdf]

ST1. GScore and BFE values of structurally distinct HDACi against HDAC2

**HDAC Inhibitor**	**Target HDAC**	**GScore**	**$\Delta G_{binding}$ (kcal/mol)**
LAQ824	HDAC2	-10.74	-67.7911
CRA-024781		-9.88	-53.2132
LBH-589		-9.88	-55.6214
HC-toxin		-6.64	-79.5371
Valproic acid		-3.61	-9.09224
