# Supplementary material for: Combinatorial In Silico Strategy towards Identifying Potential Hotspots during Inhibition of Structurally Identical HDAC1 and HDAC2 Enzymes for Effective Chemotherapy against Neurological Disorders
Source: Front Mol Neurosci. 2017 Nov 9;10:357. doi: 10.3389/fnmol.2017.00357 (PMC5684606; doi:10.3389/fnmol.2017.00357)
Supplement: Supplementary file 5 [file Table2.pdf]

ST2. GScore and BFE values of structurally distinct HDACi against HDAC1

HDAC Inhibitor	Target HDAC	GScore	$\Delta G_{binding}$ (kcal/mol)
LAQ824	HDAC1	-9.4	-63.6098
CRA-024781		-8.7	-40.8433
LBH-589		-9.51	-54.0967
HC-toxin		-6.65	-73.5482
Valproic acid		-2.48	37.09804
